# The Anatomy of a Hybrid In-Person and Virtual Sexual Health Clinic in Oncology

**DOI:** 10.3390/curroncol30020184

**Published:** 2023-02-17

**Authors:** Andrew Matthew, Steven Guirguis, Taylor Incze, Elisa Stragapede, Sarah Peltz, Gideon Yang, Leah Jamnicky, Dean Elterman

**Affiliations:** 1 Department of Surgical Oncology, Princess Margaret Cancer Centre, University Health Network, 700 University Avenue, 6th Floor, Toronto, ON M5G 1Z6, Canada; 2 Division of Urology, Department of Surgery, Mackenzie Health, Richmond Hill, ON L4C 4Z3, Canada; 3 NexJ Health, Inc., Toronto, ON M4N 3N1, Canada; 4 Division of Urology, Department of Surgery, University Health Network, University of Toronto, Toronto, ON M5T 2SB, Canada

**Keywords:** sexual health, cancer survivorship, sexual dysfunction, biopsychosocial, oncosexology, cancer rehabilitation

## Abstract

Sexual health is compromised by the diagnosis and treatment of virtually all cancer types. Despite the prevalence and negative impact of sexual dysfunction, sexual health clinics are the exception in cancer centers. Consequently, there is a need for effective, efficient, and inclusive sexual health programming in oncology. This paper describes the development of the innovative Sexual Health Clinic (SHC) utilizing a hybrid model of integrated in-person and virtual care. The SHC evolved from a fusion of the in-person and virtual prostate cancer clinics at Princess Margaret. This hybrid care model was adapted to include six additional cancer sites (cervical, ovarian, testicular, bladder, kidney, and head and neck). The SHC is theoretically founded in a biopsychosocial framework and emphasizes interdisciplinary intervention teams, participation by the partner, and a medical, psychological, and interpersonal approach. Virtual visits are tailored to patients based on biological sex, cancer type, and treatment type. Highly trained sexual health counselors facilitate the virtual clinic and provide an additional layer of personalization and a “human touch”. The in-person visits complement virtual care by providing comprehensive sexual health assessment and sexual medicine prescription. The SHC is an innovative care model which has the potential to close the gap in sexual healthcare. The SHC is designed as a transferable, stand-alone clinic which can be shared with cancer centers.

## 1. Introduction

Sexual health is compromised by the diagnosis and treatment of virtually all cancer types [1]. The nature of sexual dysfunction (SD) can differ by type of cancer and treatment but invariably involves biological, psychological, and interpersonal factors that negatively affect the patient’s overall health-related quality of life [2]. Despite the gravity of SD and the associated psychosocial sequelae, sexual health clinics are the exception in cancer centers worldwide [3,4]. This considerable gap in cancer care forms the basis for the need for sexual healthcare that is accessible and affordable without compromising effective personalized care delivery.

### 1.1. Prevalence and Severity of Impact on Health-Related Quality of Life

#### 1.1.1. Impact on Physical Wellbeing

Over 50% of cancers diagnosed in Canada are breast and pelvic cancers [5]. Of these cancers, 30% of colorectal, 73% of breast, and 90% of prostate/gynecological cancer survivors experience long-term SD. Additionally, 20% of non-breast/non-pelvic cancer survivors report SD that interferes with their overall quality of life. Ovarian and cervical cancer survivors report hot flashes, low desire, vaginal dryness, vaginal stenosis, atrophy, inflammation, discomfort, and pain during sex [6,7,8,9]. Patients undergoing treatment for prostate and testicular cancer can experience erectile dysfunction (ED), loss of desire, fatigue, hot flashes, and loss of satisfaction [2,9,10,11]. In non-genitourinary cancers, such as head and neck cancers, loss of saliva, disfigurement, and fatigue can all have an impact on sexual health and patient quality of life [12].

#### 1.1.2. Impact on Psychological Wellbeing

Although selected sexual side-effect profiles are short-term, the majority of survivors experience long-term sexual health concerns. The chronicity of the physical impact leaves patients vulnerable to psychological morbidity, including distress, depression, anxiety, and loss of self-esteem [13]. During cancer treatment, changes in body shape, scars, and hair loss can cause patients to feel less attractive or desirable and consequently experience less sexual desire themselves [14,15]. For some prostate cancer survivors, ED has been described as stripping them of their identity, sex life, and relationship [16]. Additionally, patients suffering from ED can experience performance anxiety which can interfere with adherence to pro-erectile therapy [17]. 

#### 1.1.3. Impact on Interpersonal Wellbeing

Patients with SD report reductions in overall relationship intimacy and satisfaction [17]. Physical changes in sexual functioning can cause both male and female cancer survivors to avoid sexual intimacy with their partners. This behavior can lead to decreased relationship satisfaction, which in turn can lead to further sexual dysfunction for the couple [17,18]. In a study of cervical cancer survivors and their partners, many partners described that changes in sexual health after treatment (e.g., vaginal dryness, pain during intercourse, sexual dysfunction) contributed to interpersonal problems and emotional distancing from their patient-partners [15]. 

#### 1.1.4. Barriers to Sexual Healthcare in Oncology

Despite ongoing research and documentation of the physical, psychological, and interpersonal burden of sexual dysfunction, the provision of sexual healthcare in oncology is uniformly inadequate. Research demonstrates that this gap in care can be attributed to three main factors: (1) Poor Accessibility and Access Disparity—access to care is a challenge given that most cancer centers do not offer sufficient sexual healthcare [3,4]. Poor accessibility is further exacerbated by geographical disparity. Compared to their urban counterparts, rural oncology settings often receive less funding to support specialized services, such as sexual healthcare [19]; (2) Lack of Oncologist Training and Time to Provide Sexual Healthcare—lack of communication between clinician and patient has been consistently identified as a barrier to accessing sexual healthcare [20]. The main barriers to practitioner-initiated conversations about sexual healthcare include a lack of appropriate training, resources, and time [4]. Additionally, oncologists report feeling even less prepared for understanding sociocultural factors affecting sexual health and knowing the unique care needs of LGBTQ2+ cancer survivors [21]; and (3) Lack of Financial Resources—for many reasons, including an aging population, healthcare systems have experienced significant financial strain over the past two decades [22], and the COVID-19 pandemic has contributed to even more fiscal pressure on hospitals worldwide [23,24]. As hospital budgets become increasingly inadequate for the provision of basic care, specialized sexual healthcare is less likely to receive initial and sustainable funding.

In summary, the severity of the impact of sexual dysfunction combined with the complexity of barriers to care within an already financially strained healthcare system underpins the lack of sexual health clinics in cancer centers worldwide. Consequently, there remains an exigent need for effective, efficient, and inclusive sexual health programming in oncology. This paper describes the newly developed Princess Margaret Cancer Centre (Toronto, Canada) Sexual Health Clinic (in collaboration with NexJ Health Inc.) that utilizes an innovative, hybrid model of integrated in-person and virtual care. The goal of the SHC is to assist patients and their partners in achieving optimal sexual function, satisfaction, and interpersonal intimacy post-cancer treatment. A core value of SHC is to provide equitable, inclusive care in an environment that fosters openness and sensitivity to racial, cultural, sexual, and gender diverse populations.

## 2. Materials and Methods

### Previous Work

In 2009, our team developed the Prostate Cancer Rehabilitation Clinic (PCRC), which was made available to all men who consented to a radical prostatectomy at the Princess Margaret Cancer Centre in Toronto, Canada. The PCRC was a structured, manual-enhanced sexual rehabilitation clinic for prostate cancer survivors and their partners. In 2012, the PCRC was introduced into usual care with a patient “opt-out” option. A PCRC program evaluation revealed significant patient interest and uptake [25]. In comparison to patient experience before the implementation of the PCRC, PCRC patients reported improved biomedical and psychosocial outcomes, and exceptional satisfaction scores in care provision [10]. In 2019, the TrueNorth SHAReClinic was introduced as an alternative option to the PCRC. The TrueNorth SHAReClinic is a facilitated virtual clinic containing over 200 pages of expert-informed content, 22 videos (physician, patient, and partner), and several specialized, virtual self-management features. Evaluation of the TrueNorth SHAReClinic revealed 71% patient engagement at 1-year follow-up, substantial patient activity on the platform, and non-inferior sexual health outcomes compared to “best practice” and the scientific literature [26]. Combined, the PCRC and the TrueNorth SHAReClinic have provided treatment to over 2500 patients. Currently, the TrueNorth SHAReClinic is embedded as usual care in five leading Canadian cancer centers.

Overall, the results from the PCRC and the TrueNorth SHAReClinic emphasize the importance of sexual healthcare and support the expansion of innovative models of sexual healthcare to other oncology populations. Accordingly, the Princess Margaret Sexual Health Clinic (SHC) evolved from a fusion of the Prostate Cancer (Sexual) Rehabilitation Clinic at Princess Margaret (in-person) and the Movember TrueNorth Sexual Health and Rehabilitation e-Clinic (virtual) for prostate cancer patients. The resultant hybrid care model was adapted to include six additional cancer sites (cervical, ovarian, testicular, bladder, kidney, and head and neck) with plans to expand to all cancer sites. The SHC is theoretically founded on a biopsychosocial framework and emphasizes interdisciplinary intervention teams, active participation by the partner, and a broad-spectrum medical, psychological, and interpersonal approach. Clinical and patient-reported outcome (PRO) measures are serially collected via the SHC virtual portal to assess quality assurance and effectiveness. The launch of the SHC utilized a Hybrid Type 3 implementation methodology to ensure seamless integration into the patient workflow across the cancer sites of a high-volume cancer center.

## 3. Results

### 3.1. The Sexual Health Clinic (SHC)

#### 3.1.1. Patient Population

The SHC provides care to patients (and partners) with prostate, testicular, cervical, ovarian, bladder, kidney, and head and neck cancers. These cancer sites were chosen for their diversity in age, biological sex, type of sexual dysfunction, and pelvic and non-pelvic concerns. Successful treatment across these diverse presentations will allow for easy expansion to all cancers in the future. 

#### 3.1.2. Clinical Visiting Program

**Figure 1:**
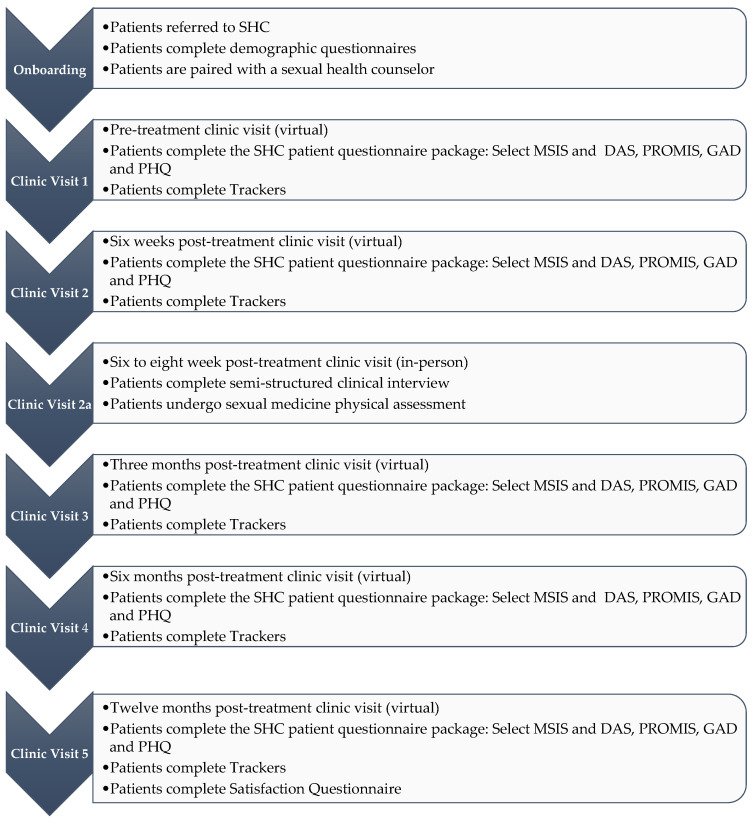
SHC Visit Schedule.

The SHC visit schedule includes a virtual clinic visit at pre-treatment (T1) and 6 weeks (T2), 3 months (T3), 6 months (T4), and 12 months (T5) post-cancer treatment. The virtual visits are facilitated by highly trained sexual health counselors (SCs) who provide education, support, and guidance via SHC platform-based asynchronous and synchronous chat, telephone, and/or videoconference. At 6–10 weeks post-cancer treatment (T2a), patients/couples also attend an in-person clinic where they are seen by an interdisciplinary team for sexual medicine assessment and prescription (note: additional in-person follow-up visits are provided as needed). The visit type and schedule are designed to maximize upfront patient education (T1), normalize treatment impact (T2), allow for early medical examination and prescription of sexual medicine (T2a), and provide long-term sexual rehabilitation (T3, T4, T5) (see Figure 1).

#### 3.1.3. Assessment

The SHC patient sexual health assessment is a three-step process comprising of a semi-structured clinical interview, completion of a standardized questionnaire package, and a physical exam. The semi-structured interview is performed by SCs during the patient’s first in-person visit (T2a). The comprehensive clinical interview documents the patient’s (1) medical and psychiatric history, cancer type and treatment, gender, and sexual orientation; (2) previous and current use of sexual medicine or devices; (3) concerns regarding sexual desire, arousal, plateau, orgasm, body image, pain, and satisfaction; and (4) concerns regarding relationship satisfaction and intimacy. The SHC patient questionnaire package includes the Miller Social Intimacy Scale (MSIS)—only four items, the Dyadic Adjustment Scale (DAS)—only one item, the Patient-Reported Outcomes Measurement Information System (PROMIS)—Full Profile Sexual Function and Satisfaction, the Generalized Anxiety Disorder-7 (GAD-7), the Patient Health Questionnaire-9 (PHQ-9), and the Patient Satisfaction Questionnaire. Additionally, patients are prompted to regularly complete sexual functioning trackers (Likert scale 1-low to 10-high) including sexual desire/interest, erection firmness (male), lubrication (female), sexual activity in the last month, sexual satisfaction, intimacy (couple), and body image concerns. The questionnaires are completed by patients at the end of each virtual care visit, while the trackers are completed per patient preference, with a minimum of once per virtual care visit. Finally, during the first in-person visit, if applicable, the patient will undergo a sexual medicine physical assessment. Combined, the SHC assessment is used to guide the development of a personalized biopsychosocial treatment plan.

#### 3.1.4. Intervention

The SHC intervention is theoretically founded on a biopsychosocial framework combined with an interdisciplinary provision of care. The intervention consists of three major components: virtual access to education modules; virtual care facilitation by a sexual health counselor; and in-person clinic visits with an interdisciplinary sexual health team. 

#### 3.1.5. Educational Modules

Patient/couple experience is tailored through the assignment of specific education modules based on the patient’s cancer type, treatment type, biological sex, and relationship status (single or coupled). A total of eighty-one multi-modal education modules, inclusive of figures, photos, videos, and animations, were created specific to the physical, psychological, and interpersonal impacts of cancer and its treatment on sexual health (Table 1: Education Module Menu). Additionally, patients can further customize their experience by adding “patient preference” modules to their virtual experience (e.g., Unique Needs of LGBTQ2+). All education modules are founded based on internationally recognized guidelines, empirical research, and expert opinion. Combined with the educational modules, the virtual platform offers access to symptom trackers and goal-setting strategies to empower participant self-management. Finally, the online clinic also features a professionally curated, digital health library including electronic books, relevant leading articles, and videos on sexual health and rehabilitation. 

Accounting for the seven cancers, the biological sex of the patient, and the cancer treatment options, a total of 32 unique patient streams were determined (e.g., a male bladder cancer surgery patient stream, a female bladder cancer surgery patient stream, a male bladder cancer surgery plus chemotherapy patient stream, etc.). Using the Education Module Menu, education modules were embedded into each of the 32 unique patient streams based on known relevance to a patient’s experience in that particular stream. For example, see Table 2 comparing the stream of a male bladder cancer surgery patient and a testicular cancer surgery patient.

#### 3.1.6. Virtual Sexual Health Counselors

In an effort to “humanize the technology” and promote patient engagement [26], the SHC virtual care platform is facilitated by sexual health counselors via chat, telephone, or videoconference. The SHC counselors provide an additional layer of personalization through the provision of psychosexual education/guidance, supportive counseling, and motivational strategies to foster self-management and SHC-treatment adherence. Training for our SHC counselors involves successful completion of the Sexual Health in Cancer Part I and Sex Counselling in Cancer Part II courses offered through the de Sousa Institute (https://www.desouzainstitute.com accessed on 13 January 2023). The SHC counselors must also complete specialized training in virtual-based communication and documentation, and platform-based features designed to enhance participant–counselor interaction (e.g., participant engagement and tracker monitoring). Additionally, the SHC counselors receive applied clinical training through SHC Case Rounds and direct supervisor–counselor shadowing during in-person clinic patient encounters.

#### 3.1.7. Face-to-Face Clinical Visits

Patients/couples attend an in-person clinic where they are seen by an interdisciplinary team inclusive of urologists, psychologists, nurses, and SCs. During their clinic visit, patients undergo both a physical examination for presenting sexual dysfunction and a psychosexual assessment for related psychosocial concerns. Where appropriate, clinic visits can also be used for in-person training in sexual medicine prescription such as intracavernosal injection training or vaginal dilator therapy. As well, the SC can schedule additional in-person follow-ups for patients requiring changes to their sexual medicine regimen, medical examination, and sexual medicine prescription. 

#### 3.1.8. Quality Assurance and Research Program

Embedded in the SHC is a quality assurance, improvement, and research program. At the core of this program is the SHC database (SHC-DB), an active database in which PRO data is serially collected, scored, and uploaded directly from the SHC platform. The SHC-DB includes data fields specific to patient demographics, patient engagement (e.g., rates of enrolment, attrition, completion), and clinic evaluation (sexual health questionnaires/trackers). The research program is designed to measure patient engagement, evaluate the effectiveness of SHC, and provide a ‘real-world laboratory’ for conducting biomedical and psychosocial research in sexual medicine. 

### 3.2. SHC Implementation

Implementation of SHC is guided by the Quality Implementation Framework and includes (1) identification of unique implementation factors within host sites, (2) creation of a systematic structure for implementation, (3) allowance for ongoing structure evolution, and (4) development of improvement strategies for future applications. In this regard, our team identified “site champions” for each of the seven cancer sites. The site champions participated in three brainstorming sessions designed to tailor integration of the SHC into patient workflow and clinic protocol. Additional key site group/clinic personnel were also interviewed to further explicate approaches to SHC integration into patient and practitioner experience. In total, 20 interviews were performed, and a pragmatic qualitative analysis of the interview transcripts is currently proceeding. To further enhance the interview data, a clinic-embedded research assistant is also documenting clinic-based enablers and barriers to successful implementation. The SHC will employ known patient-based engagement approaches for online healthcare services including establishing product legitimacy and security, usability testing and enhanced functionality, participant and SC platform training, SC facilitation, information tailoring and guided usage, patient and HCP feedback, and automated reminder features. Combined, this data collection process is helping to inform the evolution of successful SHC-integration strategies that serve to close gaps in sexual healthcare across cancer sites and within patient experience.

## 4. Discussion

The SHC offers care innovation that has the potential to overcome pervasive barriers in sexual healthcare (such as poor accessibility, financial restraint, and lack of oncologist time and specialized training) without compromising treatment adherence and effectiveness. The SHC employs a virtual dominant in-person less-dominant model of care. This design strategically combines the accessibility and efficiency benefits of digital healthcare with the personalization of face-to-face contact during in-clinic visits. Additionally, SHC utilizes virtual-counsellor facilitation and treatment tailored to maintain engagement and enhance effectiveness. 

### 4.1. SHC and Accessibility

Residents in rural areas face more difficulties accessing health care compared to their urban counterparts [27]. In general, rural residents have direct access to a much smaller number and scope of health services and providers than urban residents [28,29,30]. As a result, patients in rural locations can experience geographical inequities in accessing specialized care. Digital health interventions offer solutions to geographical health disparity via efficient, accessible, and scalable care pathways. Internet penetration in North America is believed to be over 90% [31]. Additionally, the COVID-19 pandemic stimulated technology expansion and patient acceptability of digital health intervention. The SHC virtual care-dominant approach serves to alleviate geographic disparity concerns through enhanced patient access and reductions in the need for patients to physically attend regular follow-up clinic appointments. Additionally, the scalability of digital health solutions makes SHC particularly well suited for reducing health disparities both within and across countries.

### 4.2. SHC and Affordability

Digital health interventions are generally considered to be time and cost efficient in comparison to traditional care pathways [32]. In Canada, between 2005 and 2015, innovations in digital health produced approximately CAD 16 billion in efficiencies [33] In 2017, the US FDA published the Digital Health Innovation Action Plan to encourage technology innovation in digital health [34]. Traditional survivorship care, inclusive of sexual health care, relies on in-person, structured visit schedules. In-person clinic visits incur costs to the host institution in the form of space, clinic staff wages, and incidental costs. The SHC utilizes a targeted approach to in-person care such that only patients requiring medical examination or initiation of sexual health medicine are seen in clinic while the majority of follow-up care is virtual. The cost effectiveness of a 1 to 5 ratio of in-person to virtual visits helps to ensure sustainability during times of significant fiscal restraint.

### 4.3. SHC and Oncologist Training and Time

Although oncologists recognize sexual health as an important part of their patients’ quality of life, they report feeling restricted by lack of training and lack of in-clinic time to adequately treat their patients’ sexual health concerns [31,35,36]. Unfortunately, this can lead to decreased physician–patient communication about the sexual implications of cancer treatment [37]. Given these limitations, it is likely necessary to provide care external to oncology clinics in order to optimally treat patient sexual health concerns. By providing a patient resource for specialized sexual healthcare external to busy oncology clinics, the SHC relieves oncologists from the pressure to provide sexual healthcare during busy clinic periods. Additionally, having the SHC resource available may also serve to increase the likelihood that physicians will inquire about their patients’ sexual health knowing that they can make an appropriate referral. 

### 4.4. SHC and Digital Health Engagement and Effectiveness

Although there are clear accessibility and affordability benefits associated with digital health, high dropout rates and/or poor patient adherence to digital interventions for health-related behaviors can compromise intervention effectiveness [38,39]. Core considerations for successful digital healthcare must include strategies to encourage initial and ongoing patient engagement. The SHC relies on engagement strategies that were determined to be successful in the TrueNorth SHAReClinic [25]. Specifically, the SHC relies on digital health facilitation, via virtual sexual health counselors, as a key feature in achieving engagement. By enhancing engagement, the SHC-care model allows for increased treatment “dose” and improved effectiveness. Similarly, the SHC provides for treatment tailoring [27,30] through obtaining relevant patient-specific variables that are entered into digital algorithms to organize information and management strategies specific to patient experience. Tailoring is further augmented by guiding patient experience using structured visits and care pathways [40]. Finally, regular and ongoing patient-reported symptom trackers are used to support self-management as an integral part of the SHC-intervention protocol. By targeting patients’ physical, psychological, and relational sexual health concerns, the SHC avoids the pitfall of a one-size-fits-all approach that is unlikely to be effective given the breadth and complexity of sexual dysfunction in oncology.

## 5. Conclusions

Hybrid digital-inperson healthcare models of care can provide increased access/reach, affordability, tailoring of care, cost-effectiveness, and scalability. The SHC is specifically designed to be a transferable, stand-alone clinic which can be efficiently and effectively shared with cancer centres and other institutions providing care to cancer patients.

## Figures and Tables

**Table 1:** SHC Educational Modules.

All Patients	Urinary and Bowel
Introduction Sexual Health and Wellbeing as an Important Part of Cancer Care About the SHC Understanding the Sexual Response Cycle The Sexual Response Cycle and the Impact of “Type” Cancer Treatment Fear of Passing Cancer to Partner Resuming Sexual Activity Managing Your Expectations of Recovery Process of Recovery and Anxiety About Sex Physical Affection (Holding Hands, Hugs, Touch, Kissing) Learn About Sexual Desire and Sexual Fantasy Sensate Focus Look Good Feel Better	Urinary Leaks and Incontinence Bowel Problems and Treatment Recommendations Urinary Diversions Neo-Bladder Leakage/Discharge Sexual Activity and an Ostomy Pouch Kegel Exercises
Male Sexual Function—Physical	Female Sexual Function—Physical
Erectile Dysfunction Review Course of Erectile Function Recovery Overview of Dry Orgasms Overview of Pro-Erectile Treatment Pro-Erectile Treatment Decision-Making Review Your Pro-Erectile Therapy … Again “Re-Challenging” With Pro-Erectile Therapy Orgasmic Dysfunction Ejaculatory Dysfunction Decreased Sex Drive Hormonal Changes—ADT	Vaginal Health Overview of Vaginal Reconstruction Overview of Vaginal Changes Dilation Lubrication Dyspareunia Orgasmic Dysfunction Decreased Sex Drive Hormonal Changes—Premature Menopause Having Children During Treatment
Male Sexual Function—Psychological	Female Sexual Function—Psychological
Increased Sexual Distress/Decreased Sexual Satisfaction Distress Related to Body Image RPLND Scar, Testicular Prosthesis, H&N Sexual Performance Anxiety Impact on Masculinity Beliefs Disclosure and Starting New Relationships What Is Good Sex? Why Do I Engage in Sex? Loss and Grief	Increased Sexual Distress/Decreased Sexual Satisfaction Distress Related to Body Image Impact on Femininity Beliefs Disclosure and Starting New Relationships Loss and Grief
Sexual Wellbeing and Age	Head and Neck
Critical Period in Development of Sexual Identity Sexual Wellbeing and Identity Physical Changes Due to Aging That Affect Sexual Activity	Fear of Transmitting HPV Pituitary Gland Dysfunction Tracheostomy Needing to Clear Trach G-Tube
Fertility	Social Support
How Does Cancer Treatment Affect Your Fertility—Male Fertility Preservation Options—Male How Does Cancer Treatment Affect Your Fertility—Female Fertility Preservation Options—Female	Understand the Role of Social Support
	Fatigue
	Exercise, Fatigue, and Sexual Health
All Patients	
Acceptance, Adaptation, and Continued Support Adaption to Changes in Your Sex Life Continued Communication With Your Health Coach SHC Is Not Going Anywhere	
Impact on Partner	Impact on Couple
Changes as a Partner Changes Due to Age That Affect Sexual Activity as a Partner Worry About Satisfying Your Partner Worry About Hurting Your Partner	Intimacy Intimacy as a Foundation Prism Challenging Common Interpersonal Misunderstandings Fundamental Kindness Loss of Naturalness and Spontaneity Worry About Satisfying Your Partner
Patient Preference Module	
Unique Needs of LGBTQ2+ Unique Needs of Marginalized Minorities Cultural Factors and Sexual Health Mindfulness and Sexual Wellbeing	Exercise and Sexual Health Sexual Health and Loss More on Pelvic Health Physiotherapy Premature Menopause

**Table 2:** Example of Unique Patient Streams.

Bladder Cancer	Testicular Cancer
Pre-Tx	Pre-Tx
Sexual Health and Wellbeing as an Important Part of Cancer Care	Sexual Health and Wellbeing as an Important Part of Cancer Care
About the SHC	About the SHC
Understanding the Sexual Response Cycle	Understanding the Sexual Response Cycle
The Sexual Response Cycle and the Impact of Bladder Cancer Treatment	The Sexual Response Cycle and the Impact of Testicular Cancer Treatment
Fertility and Sperm-Banking	Fertility and Sperm-Banking
Urinary Diversions	Scar and Testicular Prosthesis
Potential Changes in Your Erectile Function	Potential Changes in Your Erectile Function
Frequently Asked Questions	Frequently Asked Questions
Managing Your Expectations of Erectile Recovery	
6 weeks Post-Tx	6 weeks Post-Tx
Revisiting Managing Your Expectations	Erectile Dysfunction and Erection Pills
Overview of Pro-Erectile Therapy	Resuming Sexual Activity
Pro-Erectile Treatment Decision-Making	Exploring and Expanding Your Sexual Repertoire
Resuming Sexual Activity	Body Image—Adapting to the Removal of a Testicle
Exploring and Expanding Your Sexual Repertoire	More on Fertility
Body Image—Adapting to Your Ostomy	
3 months Post-Tx	3 months Post-Tx
Potential Changes in Your Desire	Potential Changes in Your Desire
Potential Changes in Your Orgasm	Potential Changes in Your Orgasm
Male Sexual Performance Anxiety	Male Sexual Performance Anxiety
Impact on Your Masculinity	Impact on Your Masculinity
Impact on Your Sexual Partner (Female)	Impact on Your Sexual Partner (Female)
If You Are Single … the Issue of Disclosure	If You Are Single … the Issue of Disclosure
Maintain Intimacy—Understanding Intimacy and Passion	Maintain Intimacy—Understanding Intimacy and Passion
Sensate Focus	Sensate Focus
6 months Post-Tx	6 months Post-Tx
Re-Visiting Your Pro-Erectile Therapy	“Re-Challenging” Pro-Erectile Pills
Age and Sexual Health (Female Partner)	Age, Sexual Health, and Sexual Identity
Age and Sexual Health (Patient)	Grief as a Normal Emotional Response
Grief as a Normal Emotional Response	What Is Good Sex?
What Is Good Sex?	Why Do I Engage in Sex?
Why Do I Engage in Sex?	Common Interpersonal Misunderstandings
Common Interpersonal Misunderstandings	The Big Picture: Adapting to the Diagnosis and Treatment of Cancer
	Adaptation to Changes in Your Sex Life
	Understanding Your Social Support
	Continued Communication With Your Sexual Health Coach
	SHC Is Not Going Anywhere
12 months Post-Tx	
“Re-Challenging” With Pro-Erectile Therapy	
The Big Picture: Adapting to the Diagnosis and Treatment of Cancer	
Adaption to Changes in Your Sex Life	
Understand Your Social Support	
Continued Communication With Your Sexual Health Coach	
SHC Is Not Going Anywhere	

## Data Availability

The data presented in this study are available on request from the corresponding author.
